# The Fate of a Hapten - From the Skin to Modification of Macrophage Migration Inhibitory Factor (MIF) in Lymph Nodes

**DOI:** 10.1038/s41598-018-21327-8

**Published:** 2018-02-13

**Authors:** Isabella Karlsson, Kristin Samuelsson, Carl Simonsson, Anna-Lena Stenfeldt, Ulrika Nilsson, Leopold L. Ilag, Charlotte Jonsson, Ann-Therese Karlberg

**Affiliations:** 1 Department of Environmental Science and Analytical Chemistry, Stockholm University, Stockholm, Sweden; 2 Department of Chemistry and Molecular Biology, Dermatochemistry, University of Gothenburg, Gothenburg, Sweden

## Abstract

Skin (contact) allergy, the most prevalent form of immunotoxicity in humans, is caused by low molecular weight chemicals (haptens) that penetrate *stratum corneum* and modify endogenous proteins. The fate of haptens after cutaneous absorption, especially what protein(s) they react with, is largely unknown. In this study the fluorescent hapten tetramethylrhodamine isothiocyanate (TRITC) was used to identify hapten-protein conjugates in the local lymph nodes after topical application, as they play a key role in activation of the adaptive immune system. TRITC interacted with dendritic cells but also with T and B cells in the lymph nodes as shown by flow cytometry. Identification of the most abundant TRITC-modified protein in lymph nodes by tandem mass spectrometry revealed TRITC-modification of the N-terminal proline of macrophage migration inhibitory factor (MIF) – an evolutionary well-conserved protein involved in cell-mediated immunity and inflammation. This is the first time a hapten-modified protein has been identified in lymph nodes after topical administration of the hapten. Most haptens are electrophiles and can therefore modify the N-terminal proline of MIF, which has an unusually reactive amino group under physiological conditions; thus, modification of MIF by haptens may have an immunomodulating role in contact allergy as well as in other immunotoxicity reactions.

## Introduction

About 20% of the population in the Western world have skin (contact) allergy to one or more compounds in their close environment^1^. Allergic contact dermatitis (ACD), i.e. skin inflammation and eczema, is the clinical manifestation of contact allergy and affects 5–10% of the population^2,3^. ACD is caused by T-lymphocyte mediated type IV hypersensitivity responses to antigens after skin exposure to contact allergens. Small (<1000 Da) reactive organic molecules (haptens) able to penetrate *stratum corneum* (SC), the top-most layer of the skin and covalently modify endogenous proteins are the most important contact allergens from a clinical point of view. Hapten exposure leads to activation of cutaneous dendritic cells (DCs), which migrate from the skin to the draining lymph nodes (LNs) where they present peptides (potential antigens) from hapten-modified proteins to naïve T cells; thereby, activating the adaptive (acquired) immune system. The naïve T cells that have recognized an antigen start to proliferate and differentiate into antigen-specific effector and memory T cells that circulate in the blood and lymphatic system^3–5^. Many aspects of the mechanisms underlying ACD are still unknown despite recent advances in understanding the role and interaction of different immune cells. For instance, although the hypothesis that haptens induce immune responses by modifying endogenous proteins was introduced by Landsteiner and Jacobs already in 1936^6^ the identity of these modified proteins has remained more or less unknown even until today. In particular, knowledge of the identity of hapten-protein conjugates seen as immunogenic by the immune system, i.e. that become antigens that activate naïve T cells, will increase our mechanistic understanding of contact allergy. In addition these hapten-protein conjugates could have potential as biomarkers for the development of better diagnostic tests for contact allergy and may also prove useful in the development of improved treatments. One previously unexplored approach to find potentially immunogenic hapten-modified proteins would be to identify hapten-protein conjugates in the LNs after topical administration.

The aim of the present study was to improve our understanding regarding the fate of a specific hapten after topical application and, in particular, to identify hapten-protein conjugates in LNs as these play a key role in activation of the adaptive immune system. The hapten-protein conjugates (adducts) are most often formed by reactions between electrophilic haptens and nucleophilic side chains such as cysteines (thiols) and lysines (primary amines) in skin proteins. Isothiocyanate (NCS) is an electrophilic functional group prone to react with both amines and thiols. Fluorescent isothiocyanates, e.g. fluorescein isothiocyanate (FITC), are due to their reactivity with proteins extensively used in various biological applications. In the present study mice were topically exposed to two fluorescent compounds, tetramethylrhodamine isothiocyanate (TRITC, sensitizer)^7^ and tetraethylrhodamine (Rhodamine B, non-sensitizer) and the distribution in skin and draining LNs was investigated. Microscopic examination of skin penetration of TRITC after topical application showed that the most intense fluorescence signal was detected in SC. Excision and inspection of the draining LNs from mice sensitized to TRITC revealed fluorescence in DCs but also in T and B cell populations. No fluorescence was detected in LNs in mice treated with Rhodamine B. Detection of TRITC-modified proteins isolated from LN cells, followed by identification of the most strongly TRITC-fluorescent protein, revealed TRITC bound to the N-terminal proline of the pleiotropic cytokine *macrophage migration inhibitory factor* (MIF). To the best of our knowledge, this is the first time a hapten-protein conjugate has been identified in local LNs after topical application of the hapten.

## Results

**Figure 1:**
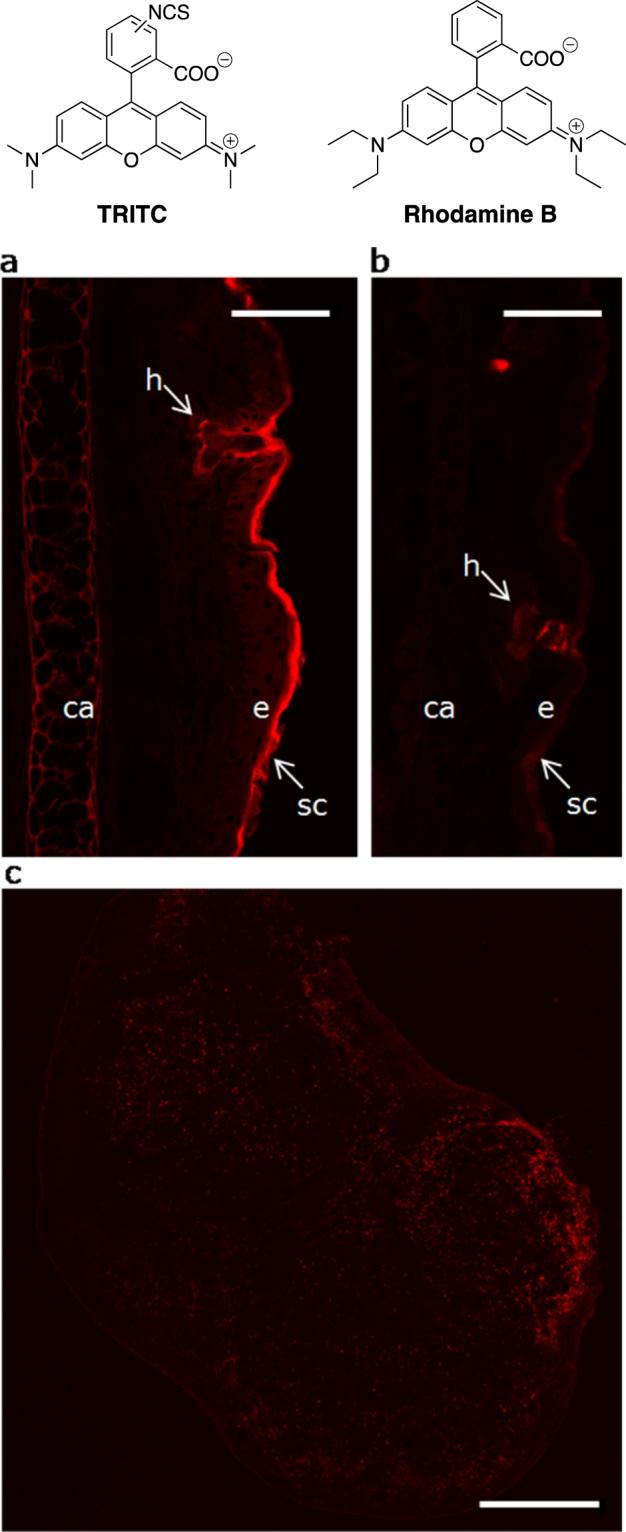
Laser scanning confocal microscopy (LSCM) images of cryosectioned tissues. Mice were exposed to 25 µl of tetramethylrhodamine isothiocyanate (TRITC) (11 mM) (**a**,**c**) or the non-sensitizing structural analogue tetraethylrhodamine (Rhodamine B) (11 mM) (**b**) on the dorsal side of each ear for three consecutive days. Eighteen hours after the last exposure, the mice were sacrificed and ears (**a**,**b**) and draining lymph nodes (**c**) were excised, snap frozen and sectioned (10 µm and 14 µm, respectively). Slides were fixed in acetone. To be able to detect Rhodamine B, the laser intensity was increased 20 times compared to the settings for TRITC. The scale bars refer to 75 µm (**a**,**b**) and 500 µm (**c**). h, hair follicle; ca, cartilage; e, epidermis; sc, stratum corneum. The structures of TRITC and Rhodamine B are shown at the top of the figure.

In the current project, the fluorescent skin sensitizer TRITC (Fig. 1) and the structurally similar compound Rhodamine B (Fig. 1), which is fluorescent but lacks the reactive NCS group of TRITC, were topically applied to the dorsum of mice ears for three consecutive days. Thereafter, the TRITC/Rhodamine B fluorescence in skin, draining LNs, LN cells and LN proteins was investigated.

### Sensitization assessment

The skin sensitizing potential of Rhodamine B was investigated in the Local Lymph Node Assay (LLNA)^8–10^, which is the recommended OECD method for assessing the skin sensitizing potential of a compound. Rhodamine B was considered a non-sensitizer in the used test concentrations (up to 5% (w/v)) as no cell proliferation was induced in the LNs (Supporting Information, Table S1). This is in contrast to TRITC, which we have previously shown to be an extreme skin sensitizer in the LLNA with an EC3 value of 0.040%^7^.

### Visualization of fluorophore distribution in mouse ears

To study the distribution of TRITC and Rhodamine B in the skin after topical *in vivo* exposure, sections of fluorophore-exposed mouse ears were investigated using laser scanning confocal microscopy (LSCM). TRITC was detected mainly in SC, in hair follicles, in the matrix of the cartilage and to a minor extent in viable epidermis (Fig. 1a). To visualize the fluorescence from the control substance Rhodamine B, the laser power had to be increased 20 times compared to the settings for the TRITC sample. The most intense Rhodamine B fluorescence was seen in hair follicles, while fluorescence in SC and cartilage was hardly detectable despite the increased laser power (Fig. 1b). The discrepancy between the distributions of the two fluorophores in the skin is likely due to the NCS group’s ability to covalently modify biomacromolecules. We have previously shown in *in vitro* studies on human skin using LSCM and two photon microscopy that FITC, structurally similar to TRITC with corresponding skin sensitizing properties^11^, accumulated in SC while fluorescein, the non-sensitizing analogue lacking the NCS group, penetrated to deeper layers of epidermis^12^.

### Visualization of fluorophores in LNs and distribution in LN cells

To further investigate the distribution of the fluorophores in mice, ear draining LNs were excised and analyzed with LSCM and flow cytometry. The presence of TRITC was clearly visualized in LN sections (Fig. 1c), whereas the negative control Rhodamine B was not observed using LSCM (data not shown). The number of LN cells was markedly increased in mice treated with TRITC and the proportion of fluorescent LN cells was estimated to on average 77% by flow cytometry (Table S2). The number and fluorescence of LN cells in Rhodamine-exposed mice was the same as for vehicle-treated mice; hence corroborating the LSCM study in which no Rhodamine B fluorescence was detected in the LN section.

**Figure 2:**
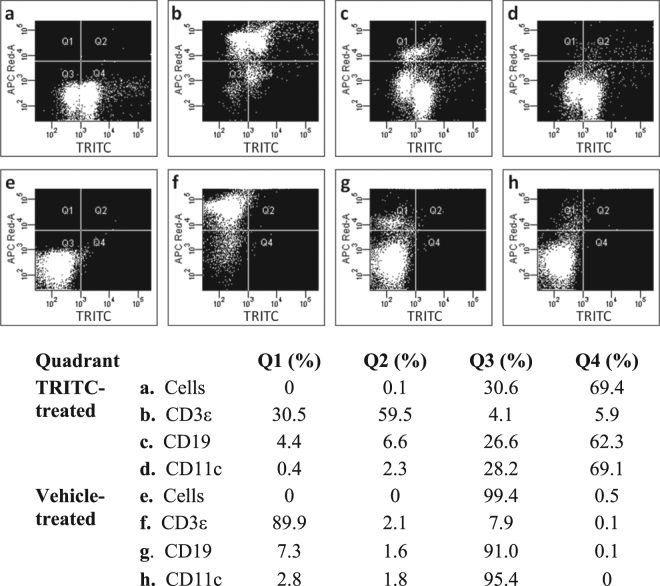
Flow cytometry analysis of lymph node cell populations. Cells from ear draining lymph nodes isolated from mice exposed to TRITC (5.6 mM in acetone:dibutyl phthalate, 1:1, 25 µl) (**a**–**d**) or vehicle (acetone:dibutyl phthalate, 1:1, 25 µl) (**e**–**h**) on both ears for three consecutive days were analyzed by flow cytometry. Lymph node cells from each treatment group were pooled before analysis. The dot plots show unstained cells (**a**,**e**) and cells stained with allophycocyanin (APC) labeled antibodies toward T cells (CD3ε; **b**,**f**), B cells (CD19; **c**,**g**), or DC (CD11c; **d**,**h**). Q1 (upper left quadrant) shows the proportion of APC positive/TRITC negative cells and Q2 (upper right) shows the proportion of double positive cells. Q3 (lower left) shows the proportion of double negative cells and Q4 (lower right) the proportion of APC negative/TRITC positive cells.

Earlier skin painting studies with TRITC have identified DCs as responsible for the accumulation of the fluorophore in LNs^13–16^. Indeed, our study shows that almost all DCs were TRITC positive (around 85%). However, our investigation analyzed different cell types and could show that T and B cells were the major cellular targets of the fluorophore (Fig. 2). While DCs constituted a minor part (2–3%) of the LN cell population, T-cells were the main cell population (around 90%) in the LNs of the TRITC exposed mice and two thirds of the T-cells were TRITC positive. B cells constituted about 10% of the LN cells and of these more than half were TRITC positive. Thus, the high yield of TRITC positive cells in the LN is unlikely to solely depend on transport of haptenated proteins/peptides from the skin by LN immigrating DCs. Studies of other fluorescent isothiocyanates, by us^17^ and others^18,19^, have shown that in addition to the relatively slow cell-mediated transport of these compounds to the local LNs by DCs, they are also transported as free haptens or soluble hapten-protein complexes via the lymph and blood to the secondary lymphoid organs. In the LNs, these soluble haptens/hapten-protein conjugates might be captured by antigen-presenting cells such as resident DCs, macrophages, or B cells.

### Hapten-modified proteins in LNs

**Figure 3:**
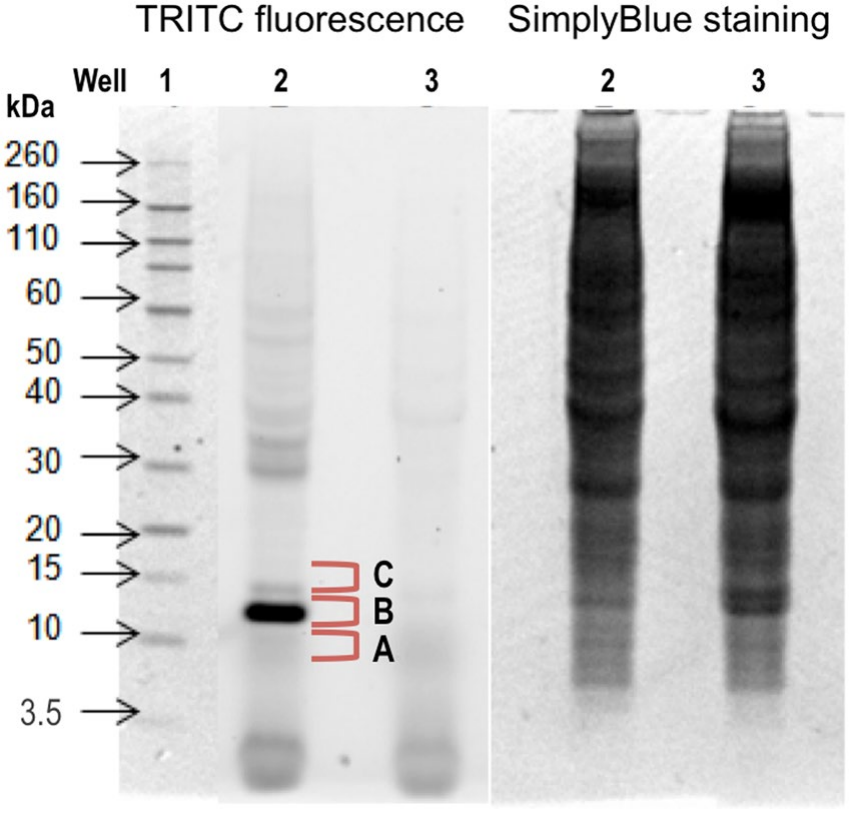
Gel separation of proteins from lymph node cells. Lymp node cell proteins were isolated from pooled lymph node cells from mice exposed to vehicle (acetone:dibutyl phthalate, 1:1) or TRITC (5.6 mM) on the dorsum of the ears for three consecutive days. Proteins were separated using 1D SDS-PAGE and TRITC fluorescence (panel 1) was visualized using a fluorescence scanner with a GreenLED 528 nm laser with a bandpass filter of 605 ± 50 nm. Each well was loaded with 100 µg of lymph node protein from TRITC (well 2) or vehicle (well 3) exposed mice. The Novex Sharp pre-stained standard was used as a molecular standard (well 1). The total protein contents in wells 2 and 3 were visualized with SimplyBlue staining (panel 2).

To identify which proteins in the LN cells that were modified by TRITC, the following experiments were conducted. Lysed single-cell suspensions from LNs of TRITC- or vehicle-treated mice were separated using gel electrophoresis. Fluorescent proteins were visualized by scanning at 532 nm (excitation)/580 nm (emission) (Fig. 3). One strong fluorescent protein band and several weaker fluorescent protein bands were visualized in the gel separation of LN proteins from TRITC exposed mice (Fig. 3, panel 1 – TRITC fluorescence, well 2). No fluorescent bands could be detected in the gel separation of LN proteins from the vehicle-exposed mice (Fig. 3, panel 1 – TRITC fluorescence, well 3).

**Figure 4:**
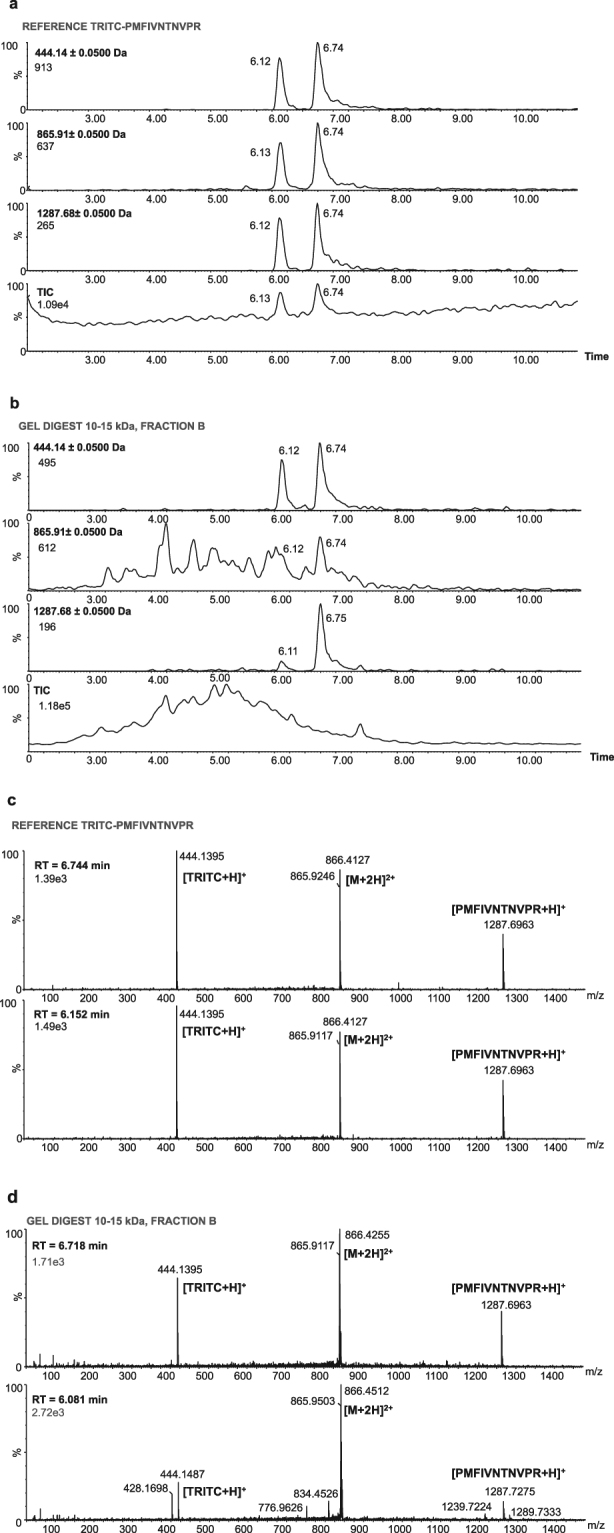
Chromatogram and spectra of TRITC-modified MIF chromatograms and spectra of the reference compound (TRITC bound to the N-terminal MIF peptide PMFIVNTNVPR) and fraction B from lymph node cell proteins isolated from TRITC-exposed mice and separated with 1D SDS-PAGE. The selected bands (see Fig. 3) were excised, digested and analyzed with UPLC-qToF. A reference compound was prepared to confirm the identity of the peptide. The qToF was operated in positive resolution mode with electrospray ionization. A qToF ms/ms method was used where the fixed mass was set to 865.9, the ms^2^ was set to 50–1500 m/z and the collision energy ramp was from 15 eV to 40 eV. The extracted chromatograms (**a**,**b**) correspond to the TRITC-fragment [m/z = 444.14, singly charged ion], the MIF peptide-fragment [m/z = 1287.68, singly charged ion] and the full TRITC-MIF conjugate [m/z = 865.91, doubly charged ion]. (**a**) Chromatogram of reference compound, (**b**) chromatogram of fraction B, (**c**) spectrum of reference compound and (**d**) spectrum of fraction B.

The most intense fluorescent band (10–15 kDa) was excised, digested and analyzed with nanoflow liquid chromatography (nanoLC) and hybrid linear ion-trap Fourier-transform ion cyclotron resonance (FT-ICR) tandem mass spectrometry (MS/MS). With the use of the software MASCOT the acquired tandem mass spectra of the tryptic peptides were searched against the Swiss prot database, which resulted in the identification of 10 different proteins (Table 1). This procedure only identified known tryptic peptides and matched them with the protein database, i.e. although modified peptides could not be identified with this method, it is still possible that one or several of these proteins are TRITC-modified. Of the identified proteins, MIF was of particular interest for two main reasons, 1) MIF is known to play an important roll in inflammatory and immune diseases^20–24^ and 2) we have previously shown that TRITC is very reactive toward N-terminal prolines and that the formed conjugates (TRITC-thiourea) are stable adducts^7^. MIF has an N-terminal proline with a remarkably low pKa of 5.6^25^, which turns the amino group into an unusually strong nucleophile under physiological conditions. Interestingly, reports in the literature describe specific modification of MIF by other compounds with an isothiocyanate functionality^26–28^. Western blot immunostaining with a MIF polyconal antibody was used to verify the co-localization of MIF with the most intense TRITC fluorescent band (Figure S1). To confirm that TRITC is actually bound to MIF, the suspected TRITC-modified MIF peptide (TRITC-PMFIVNTNVPR) was generated and used as a reference compound in a targeted screening for TRITC-MIF conjugates in the LN fractions using ultra performance liquid chromatography (UPLC) and electrospray (ESI) quadrupole time-of-flight (qToF) MS/MS. The analyses showed that TRITC bound to the N-terminal proline of MIF was present in the B fraction (Fig. 3) of the 10–15 kDa section of the LN proteins from TRITC-treated animals. The developed MS/MS method uses a fixed mass (in the first quadrupole) of 865.9 for TRITC-MIF and 644.3 for non-modified MIF; both masses correspond to doubly charged molecular ions. The displayed chromatograms in Fig. 4a (reference compound) and Fig. 4b (B fraction) represent extracted masses corresponding to the TRITC-fragments (m/z = 444.14, singly charged ion), the MIF peptide-fragment (m/z = 1287.68, singly charged ion), as well as the full TRITC-MIF conjugate (m/z = 865.91, doubly charged ion). In Fig. 4c,d, the spectra from the reference compound and the corresponding peaks in fraction B are shown. Two peaks with different retention times, but the same exact mass and fragmentation pattern were seen. In our study the mice were treated with commercially obtained TRITC consisting of a mixture of six isomers. The reference TRITC-peptide (TRITC-PMFIVNTNVPR), which was prepared from the same mix of TRITC isomers, gave the same two peaks as those observed in the B fraction of the 10–15 kDa sample (Fig. 4).

**Table 1:** Identification of potential TRITC-labeled lymph node cell proteins using nanoLC-MS/MS.

Uniprot number	Protein name	MW (kDa)	PI(calculated)	Score	No of peptides
P62962	Profilin-1	14.9	8.28	674.16	4
P02088	Hemoglobin subunit beta-1	15.8	7.65	366.52	6
P34884	Macrophage migration inhibitory factor	12.5	7.34	230.42	2
O35215	D-Dopachrome decarboxylase	13.1	6.54	188.14	5
Q9ERR7	15 kDa Selenoprotein	17.8	5.35	183.54	3
P61979	Heterogeneous nuclear ribonucleoprotein K	50.9	5.54	178.32	2
P02089	Hemoglobin subunit beta-2	15.9	8.05	128.04	3
P61971	Nuclear transport factor 2	14.5	5.38	109.47	2
P62806	Histone H4	11.4	11.36	60.87	2
P62270	40 S Ribosomal protein S18	17.7	10.99	42.05	2

No TRITC-modified MIF peptide (TRITC-PMFIVNTNVPR) was detected in the other two fractions (A and C) of the 10–15 kDa sample. Non-modified MIF peptide (PMFIVNTNVPR) was detected in fraction A only (Figure S2). With the use of a standard curve (Figure S3), the concentration of PMFIVNTNVPR in fraction A was determined to be around 7–8 nM (see Supplementary Information). By comparison with the reference sample and the assumption that all depletion of PMFIVNTNVPR in the reference sample was due to reaction with TRITC, the concentration of TRITC-PMFIVNTNVPR in fraction B was estimated to be around 2–3 nM (see Supplementary Information). Thus, the portion of modified MIF in the LN would be around 20–30% of the total amount of MIF (non-modified MIF (7–8 nM) plus TRITC-modified MIF (2–3 nM)).

## Discussion

Although contact allergy is the most prevalent form of immunotoxicity in humans and involves about a fifth of the population in the Western world there is a remarkable lack of mechanistic knowledge. A recent review by Koppes *et al*. concludes that despite the vast amount of research into skin sensitization, promising specific biomarkers for ACD have not yet been described^29^. In particular, there is a dearth of knowledge concerning the identity of hapten-protein complexes that are immunogenic. Reactive organic chemicals can modify proteins in the skin – a feature which is crucial for the activation of the innate immune system^3–5^. However, these hapten-protein conjugates may not be identical to the antigens that activate the naïve T cells in the LNs i.e. hapten-modified proteins involved in activating the adaptive immune response. Whether the hapten-protein conjugates identified in earlier research focusing on identifying protein targets in skin^30^ and *in vitro*^31–35^, are actually involved in activation of the adaptive immune system is unknown. In the current study, the objective was to identify hapten-protein conjugates in LNs rather than in the skin with the rationale that a hapten-modified protein in the LNs may play a key role in activation of the adaptive immune system and may therefore be a biomarker for ACD.

The homotrimeric protein MIF is known to have keto-enol tautomerase activity, which is catalyzed by its N-terminal proline^36–38^. Analysis of the three-dimensional structure of MIF has shown that a solvent accessible pocket is formed around the N-terminal proline forming the catalytic site. The hydrophobic residues around the N-terminal proline in the active site lower the p*K*_a_ of the amino group to such an extent that it is unprotonated at physiological pH^25^. This makes the N-terminal proline of MIF significantly more reactive than lysine amino groups, which normally are protonated under physiological conditions. Traditionally, experiments to identify targets for haptens have mainly focused on reactivity toward cysteine and lysine and although most haptens have been shown to be more reactive toward cysteine than lysine, we as well as others have demonstrated that hapten-cysteine adducts are often unstable^7,39–41^. There are, however, some studies by us and others in which the reactivity of the peptide PHCKRM toward a number of haptens, such as isothiocyanates^7^, epoxides^42–44^, oximes^45^, and anhydrides^41^ has been investigated. These studies found that the investigated haptens formed stable adducts by covalent binding to the N-terminal proline of PHCKRM. The results from the earlier investigations are in line with the present findings; although, there are differences, especially in 3D structure, between the N-terminal proline of the linear synthetic peptide PHCKRM and the N-terminal proline of MIF situated in a catalytic pocket.

MIF is expressed by more or less all immune cells such as T cells, B cells, DCs, monocytes, macrophages, neutrophils, eosinophils, basophil and mast cells; in addition, MIF is expressed in tissues that come in direct contact with our external environment, such as the skin^22^. Thus, it is possible that MIF could be a target protein for most contact allergens, either via a direct reaction with the N-terminal proline or via a primary, unstable, cysteine binding. Further support for this hypothesis is that contact hypersensitivity toward the haptens trinitrochlorobenzene and oxazolone has been shown to be impaired in MIF deficient mice^46^. Interestingly, it has been suggested that MIF requires a co-factor, such as a substrate for a chemical reaction, in order to induce immune reactions^25^. Hence, one could hypothesize that haptens, by covalent binding to the N-terminal proline in the catalytic site of MIF, might function as such co-factors.

Another hypothesis is that TRITC or TRITC-MIF, in addition to the classic major histocompatibility complex (MHC) – T cell receptor (TCR) pathway, is able to activate T cells via the so-called p-i mechanism (pharmacological interaction with immune receptors)^47^. The p-i mechanism, which states that certain compounds can bind directly to T cell receptors (TCRs) and MHC molecules, has been suggested for T cell mediated drug hypersensitivity as well as ACD^48–51^. The p-i concept bears strong similarities to super-antigen stimulations^52^, which are caused by antigens produced by certain pathogenic viruses and bacteria. Support for the hypothesis that TRITC or TRITC-MIF can activate T cells via the p-i mechanism can be found in the literature. In a study by Diamond *et al*., FITC-conjugates were shown to activate FITC-specific T cells through direct binding to the TCRs without involvement of MHC^53^. Importantly though, the p-i concept also states that direct binding of drugs/haptens/haptenated proteins to naïve T cells is, in general, not enough to initiate an immune response. However, binding to primed T cells, which have a lower threshold than naïve T cells, could lead to further T cell stimulation and expansion^47,54^. Thus, it would appear as if the p-i effect is most important in the effector phase of ACD when memory T cells are already present. Nevertheless, TRITC-MIF binding to T cells would still occur in the sensitization phase. As T cells can bind MIF via CXCR4^55^, the large population of TRITC-labeled T- and B-cells detected in the LNs (Fig. 2) might be due to TRITC-MIF interaction with Ii, CXCR4, or any other MIF receptors that lymphocytes express.

To the best of our knowledge, this is the first time that a hapten-modified protein has been identified in local LNs after topical application. Thus, it is possible that hapten-modified MIF is a new biomarker for ACD; however, studies of other haptens would need to be conduct to investigate if hapten-modification of MIF is a general feature in contact allergy. MIF secretion has been correlated to a number of autoimmune and inflammatory diseases, including atopic dermatitis^24^, arthritis^56^, colitis^57^, sepsis^58,59^, and even various cancers and their control by the immune system^60–64^. Thus, MIF’s role as a hapten target, its potential involvement in the formation of immunogenic complexes and its immunomodulatory role in the innate and adaptive immune systems may be of significance not only in ACD but also in many other diseases. Hence, this work should provide a basis for investigation of several aspects of hapten-modification of MIF in immunotoxicity, such as: a) the role of proline in formation of immunogenic complexes, b) how administration of hapten-modified MIF, instead of hapten, would affect the potency of contact sensitization, c) where and when the MIF-hapten conjugates are formed, d) the role of different types of hapten bindings (e.g. covalent binding to proteins vs. p-i effects) and e) immunoregulation by haptenated proteins in immunotoxicity beyond contact sensitization.

## Material and Methods

### Chemicals

Acetone was purchased from Merk (Darmstadt, Germany). Tetramethylrhodamine isothiocyanate (TRITC), tetraethylrhodamine (Rhodamine B), dibutyl phthalate, phosphate-buffered saline (PBS) tablets (without Mg^2+^ and Ca^2+^), trichloroacetic acid (TCA) (>99%), dithiothreitol (DTT) (>99%), ammonium bicarbonate (99%), iodoacetamide (IAA) (>99%) and Clarion mounting medium, were purchased from Sigma-Aldrich Chemie (Steinheim, Germany). [Methyl-^3^H] thymidine was purchased from Amersham Biosciences (Buckinghamshire, UK). Peptide PMFIVNTNVPR was obtained from Peptide 2.0 (Chantilly, VA). Trypsin was purchased from Roche Applies Science (Mannheim, Germany). Trypsin/Lys-C mix was obtained from Promega (Madison, WI).

### Animals

Female CBA/Ca mice (B&K Scanbur, Sollentuna, Sweden) 8–12 weeks of age were used. The mice were housed in cages with HEPA-filtered airflow under conventional conditions in light-, humidity- and temperature-controlled rooms. The regional ethics committee, Jordbruksverket, approved all experimental protocols and the animal procedures were carried out in accordance with the approved guidelines.

### LLNA

The sensitizing potential of Rhodamine B was determined using the LLNA^8–10^ (see Supplementary Materials online for details).

### Treatment of mice and tissue collection for fluorophore distribution studies

Groups of mice were topically exposed to 25 µl of TRITC (0.25% (w/v), 5.6 mM, 12 mice or 0.50%, 11 mM, 6 mice), Rhodamine B (0.54%, 11 mM, 6 mice), or vehicle (acetone:dibutyl phtalate, 1:1, 18 mice), on the dorsum of the ears for three consecutive days. Eighteen hours after the last exposure, the mice were sacrificed and the ears and the auricular LNs were excised (see Supplementary Materials online for details).

### LSCM of ear- and LN sections

Frozen ears and LNs were sectioned into 10 µm and 14 µm thick samples, respectively, on a cryostat (Leica Microsystems, Wetzlar, Germany). Imaging of ear and LN thin sections was performed using an LSCM 510 Meta system (Zeiss, Jena, Germany), at the Centre for Cellular Imaging, University of Gothenburg, Sweden (see Supplementary Materials online for details).

### Detection of fluorescent draining LN cells using flow cytometry

Single cell suspensions of LN cells were washed with PBS and diluted to 10^6^ cells ml^−1^ in PBS. The proportion of fluorescent LN cells from mice exposed to TRITC (11 mM) was compared to the proportion in mice exposed to Rhodamine B (11 mM). The distribution of TRITC in different LN cell populations from mice exposed to TRITC (5.6 mM) was investigated using typical markers for B-cells (CD19), T-cells (CD3ε) and dendritic cells (CD11c). Cells from vehicle-treated mice were used as a negative control. The analyses were conducted with a FACSArray flow cytometer (BD Biosciences) equipped with a 532 nm excitation laser (see Supplementary Materials online for details).

### Detection of TRITC-modified proteins in LN cells

LN single cell suspensions from TRITC- or vehicle- exposed mice were pooled group-vise and lysed using Pierce IP Lysis buffer (Fischer scientific) supplied with Halt Protease Inhibitor Cocktail (Fischer Scientific), according to the manufacturer’s instructions and stored at −80 °C until used for protein analyses. Protein contents were estimated using Pierce BCA protein assay reagent kit with BSA as standard (Fischer Scientific) according to the manufacturer’s instructions. In accordance with Invitrogen NuPAGE gel electrophoresis guide, proteins (100 μl/well) were separated on a 1 mm 4–12% gradient Bis-Tris gel (Invitrogen NuPAGE) separating 2–200 kDa proteins. Proteins were dissolved in Invitrogen NuPAGE LDS sample buffer containing DTT (50 mM) and each well was loaded with 100 µg protein. The gel was run in darkness for 37 min at 200 V in a Novex mini cell electrophoresis system (Invitrogen) using Novex Sharp pre-stained standard (Invitrogen) as reference. Fluorescent proteins were detected using a fluorescence scanner, Versa Doc 4000 (Bio-Rad) equipped with a GreenLED, 528 nm laser and a bandpass filter (605 ± 50 nm) and analyzed with the software Quantity One 4.6.9. To confirm an even protein loading in the wells, the gel was stained using SimplyBlue SafeStain (Invitrogen).

### Protein identification using nanoLC/ESI-MS/MS

The gel piece between 10–15 kDa (containing the most intense TRITC-fluorescent band) was excised and in-gel digestion, including reduction with DTT and alkylation with IAA, was performed using Trypsin (Roche Applies Science) according to the manufacturer’s instructions. Extracted peptides were lypholized using a DNA120 SpeedVac (Thermo Scientific) and reconstituted in 0.1% formic acid (aq.). The sample was analyzed on a nanoLC system coupled to a hybrid linear ion-trap FT-ICR mass spectrometer equipped with a 7 T magnet (LTQ-FT, Thermo Scientific). The nanoLC system was equipped with an HTC-autosampler connected to an Agilent 1100 binary pump on which the peptides were trapped on a precolumn (45 × 0.075 mm i.d.) and separated on a reversed phase column 200 × 0.050 mm, both packed in-house with 3 µm Reprosil-Pur C_18_-AQ particles. A 40 min gradient from 10% to 50% acetonitrile in water containing 0.2% formic acid was used for separation of peptides. The flow through the analytical column was 100 nl/min and the injection volume was 2 µl. The mass spectrometer was operated in data-dependent mode, automatically switching to MS/MS mode. MS-spectra were acquired in the LTQ-trap. For each scan of FT-ICR, the three most intense doubly or triply charged ions were sequentially fragmented in the linear trap by collision-induced dissociation. All the tandem mass spectra were searched by MASCOT (Matrix Science) against the Swiss prot database 57.1. The search parameters were set to: All species, MS accuracy 5 ppm, MS/MS accuracy 0.5 Da, one missed cleavage by trypsin allowed, fixed modification of propionamide modification of cysteine and variable modification of oxidized methionine. For protein identification the minimum criteria were two tryptic peptides matched at or above 95% level of confidence.

### Detection of the potential TRITC target-protein MIF with immunostaining

The approximate co-localization of MIF with the TRITC-fluorescent protein band in gel-separated proteins derived from LN cells was confirmed with western blot (see Supplementary Materials online for details).

### Detection of TRITC-modified and non-modified MIF-peptide (PMFIVNTNVPR) in LN fractions using UPLC-qToF

To identify TRITC-bound MIF and non-modified MIF in the LN single cell suspensions from TRITC-treated mice, the 10–15 kDa section from the gel electrophoresis separation of the proteins was divided into three pieces (Fig. 3): **A** – the piece just below the fluorescent band, **B** – the fluorescent band and **C** – the piece just above the fluorescent band. In-gel digestion, including reduction with DTT and alkylation with IAA, was performed using Trypsin/Lys-C mix (Promega) according to the manufacturer’s instructions. The extracted peptides were subsequently concentrated to 30% of the volume using a stream of N_2_.

UPLC-qToF analysis was performed using an Aquity UPLC system (Waters Corporation) coupled to a SYNAPT G2 (Waters MS Technologies) orthogonal acceleration qToF mass spectrometer. The UPLC was equipped with an Aquity CSH C18 column (150 × 2.1 mm i.d., particle size 1.7 μm, Waters Corporation, Milford, MA). Mobile phase A consisted of 0.1% formic acid in water and mobile phase B of 0.1% formic acid in acetonitrile. Aliquots of 10 μl of sample or reference was injected onto the column and eluted with a flow rate of 0.1 ml/min and a column temperature of 40 °C. The gradient conditions used were: 0 min 5% B, 10 min 60% B, 11 min 95% B, 12 min 95% B. The system was equilibrated with 5% B for 3 min between each run. The qToF was operated in positive resolution mode with electrospray ionization. The nebulizer gas was set to 800 l/h at a temperature of 450 °C. The cone gas was set to 100 l/h and the source temperature to 120 °C. The capillary voltage was set to 3000 V and the sample cone voltage was set to 80 V. A ToF ms/ms method was used where the fixed mass was set to 865.9 or 644.3 m/z, the ms^2^ was set to 50–1500 m/z and the collision energy ramp was from 15 eV to 40 eV. To ensure mass accuracy, all analyses were acquired using LockSpray with Leucine-enkephaline (556.2771 m/z) as lock mass. The obtained chromatograms and MS spectra where processed with the smooth function in the MassLynx software (Waters Corporation). The window size was set to ±3 scans and the number of smooths to 5. The Savitzky Golay method was used for chromatograms and the mean method for MS spectra.

To confirm the identity of the non-modified and TRITC-modified peptides, synthetic reference compounds were used. A reference mixture was prepared by mixing 200 µl of PMFIVNTNVPR (0.5 mM in sodium phosphate buffer (100 mM) pH 7.5/methanol 1:1) with 50 µl of TRITC (3 mM in methanol) and 750 µl of sodium phosphate buffer pH 7.5, resulting in final concentrations of 0.1 mM and 0.15 mM for PMFIVNTNVPR and TRITC, respectively. The mixture was left at room temperature for 24 h followed by 10,000-fold dilution with ammonium bicarbonate (25 mM) pH 8.0. With the use of a standard curve of PMFIVNTNVPR the concentrations of PMFIVNTNVPR and TRITC- PMFIVNTNVPR in the reference sample and the gel pieces were estimated (see Supplementary Materials online for details).

### Data availability statement

The datasets generated and/or analysed are available on reasonable request.

## Electronic supplementary material

Supplementary Information

## References

[1] Thyssen JP, Linneberg A, Menne T, Johansen JD (2007). The epidemiology of contact allergy in the general population–prevalence and main findings. Contact Dermatitis.

[2] Peiser M (2012). Allergic contact dermatitis: epidemiology, molecular mechanisms, *in vitro* methods and regulatory aspects. Current knowledge assembled at an international workshop at BfR, Germany. Cell Mol Life Sci.

[3] Martin SF (2015). Immunological mechanisms in allergic contact dermatitis. Curr Opin Allergy Clin Immunol.

[4] Kaplan DH, Igyarto BZ, Gaspari AA (2012). Early immune events in the induction of allergic contact dermatitis. Nat Rev Immunol.

[5] Martin SF (2015). New concepts in cutaneous allergy. Contact Dermatitis.

[6] Landsteiner K, Jacobs J (1936). Studies on the Sensitization of Animals with Simple Chemical Compounds. Ii. J Exp Med.

[7] Karlsson I (2016). Peptide Reactivity of Isothiocyanates–Implications for Skin Allergy. Sci Rep.

[8] Basketter DA (2002). Local lymph node assay - validation, conduct and use in practice. Food Chem Toxicol.

[9] Kimber I, Weisenberger C (1989). A murine local lymph node assay for the identification of contact allergens. Assay development and results of an initial validation study. Arch Toxicol.

[10] Gerberick GF, Ryan CA, Dearman RJ, Kimber I (2007). Local lymph node assay (LLNA) for detection of sensitization capacity of chemicals. Methods.

[11] Dearman RJ, Kimber I (2000). Role of CD4(+) T helper 2-type cells in cutaneous inflammatory responses induced by fluorescein isothiocyanate. Immunology.

[12] Samuelsson K (2009). Accumulation of FITC near stratum corneum-visualizing epidermal distribution of a strong sensitizer using two-photon microscopy. Contact Dermatitis.

[13] Kissenpfennig A (2005). Dynamics and function of Langerhans cells *in vivo*: dermal dendritic cells colonize lymph node areas distinct from slower migrating Langerhans cells. Immunity.

[14] Kamath AT, Henri S, Battye F, Tough DF, Shortman K (2002). Developmental kinetics and lifespan of dendritic cells in mouse lymphoid organs. Blood.

[15] Kinnaird A, Peters SW, Foster JR, Kimber I (1989). Dendritic cell accumulation in draining lymph nodes during the induction phase of contact allergy in mice. Int Arch Allergy Appl Immunol.

[16] Cumberbatch M, Kimber I (1990). Phenotypic characteristics of antigen-bearing cells in the draining lymph nodes of contact sensitized mice. Immunology.

[17] Simonsson C, Stenfeldt AL, Karlberg AT, Ericson MB, Jonsson CA (2012). The pilosebaceous unit–a phthalate-induced pathway to skin sensitization. Toxicol Appl Pharmacol.

[18] Pior J, Vogl T, Sorg C, MacHer E (1999). Free hapten molecules are dispersed by way of the bloodstream during contact sensitization to fluorescein isothiocyanate. J Invest Dermatol.

[19] Sixt M (2005). The conduit system transports soluble antigens from the afferent lymph to resident dendritic cells in the T cell area of the lymph node. Immunity.

[20] Su H, Na N, Zhang X, Zhao Y (2017). The biological function and significance of CD74 in immune diseases. Inflamm Res.

[21] Shimizu T (2005). Role of macrophage migration inhibitory factor (MIF) in the skin. J Dermatol Sci.

[22] Calandra T, Roger T (2003). Macrophage migration inhibitory factor: a regulator of innate immunity. Nat Rev Immunol.

[23] Grieb G, Kim BS, Simons D, Bernhagen J, Pallua N (2014). MIF and CD74 - suitability as clinical biomarkers. Mini Rev Med Chem.

[24] Shimizu T, Abe R, Ohkawara A, Mizue Y, Nishihira J (1997). Macrophage migration inhibitory factor is an essential immunoregulatory cytokine in atopic dermatitis. Biochem Biophys Res Commun.

[25] Swope M, Sun HW, Blake PR, Lolis E (1998). Direct link between cytokine activity and a catalytic site for macrophage migration inhibitory factor. EMBO J.

[26] Brown KK (2009). Direct modification of the proinflammatory cytokine macrophage migration inhibitory factor by dietary isothiocyanates. J Biol Chem.

[27] Ouertatani-Sakouhi H (2009). A new class of isothiocyanate-based irreversible inhibitors of macrophage migration inhibitory factor. Biochemistry.

[28] Spencer ES (2015). Multiple binding modes of isothiocyanates that inhibit macrophage migration inhibitory factor. Eur J Med Chem.

[29] Koppes SA (2017). Current knowledge on biomarkers for contact sensitization and allergic contact dermatitis. Contact Dermatitis.

[30] Simonsson C (2011). Caged fluorescent haptens reveal the generation of cryptic epitopes in allergic contact dermatitis. J Invest Dermatol.

[31] Elbayed K (2013). HR-MAS NMR spectroscopy of reconstructed human epidermis: potential for the *in situ* investigation of the chemical interactions between skin allergens and nucleophilic amino acids. Chem Res Toxicol.

[32] Aleksic M (2007). Investigating protein haptenation mechanisms of skin sensitisers using human serum albumin as a model protein. Toxicol In Vitro.

[33] Parkinson E (2014). Stable isotope labeling method for the investigation of protein haptenation by electrophilic skin sensitizers. Toxicol Sci.

[34] Dietz L (2010). Tracking human contact allergens: from mass spectrometric identification of peptide-bound reactive small chemicals to chemical-specific naive human T-cell priming. Toxicol Sci.

[35] Jenkinson C (2010). Characterization of p-phenylenediamine-albumin binding sites and T-cell responses to hapten-modified protein. J Invest Dermatol.

[36] Rosengren E (1996). The immunoregulatory mediator macrophage migration inhibitory factor (MIF) catalyzes a tautomerization reaction. Mol Med.

[37] Sun HW, Bernhagen J, Bucala R, Lolis E (1996). Crystal structure at 2.6-A resolution of human macrophage migration inhibitory factor. Proc Natl Acad Sci USA.

[38] Lubetsky JB, Swope M, Dealwis C, Blake P, Lolis E (1999). Pro-1 of macrophage migration inhibitory factor functions as a catalytic base in the phenylpyruvate tautomerase activity. Biochemistry.

[39] Fleischel O, Gimenez-Arnau E, Lepoittevin JP (2009). Nuclear magnetic resonance studies on covalent modification of amino acids thiol and amino residues by monofunctional aryl 13C-isocyanates, models of skin and respiratory sensitizers: transformation of thiocarbamates into urea adducts. Chem Res Toxicol.

[40] Nakamura T, Kawai Y, Kitamoto N, Osawa T, Kato Y (2009). Covalent modification of lysine residues by allyl isothiocyanate in physiological conditions: plausible transformation of isothiocyanate from thiol to amine. Chem Res Toxicol.

[41] Ahlfors SR, Kristiansson MH, Lindh CH, Jonsson BA, Hansson C (2005). Adducts between nucleophilic amino acids and hexahydrophthalic anhydride, a structure inducing both types I and IV allergy. Biomarkers.

[42] Nilsson AM, Bergstrom MA, Luthman K, Nilsson JL, Karlberg AT (2005). A conjugated diene identified as a prohapten: contact allergenic activity and chemical reactivity of proposed epoxide metabolites. Chem Res Toxicol.

[43] Niklasson IB, Broo K, Jonsson C, Luthman K, Karlberg AT (2009). Reduced sensitizing capacity of epoxy resin systems: a structure-activity relationship study. Chem Res Toxicol.

[44] Niklasson IB, Delaine T, Luthman K, Karlberg AT (2011). Impact of a heteroatom in a structure-activity relationship study on analogues of phenyl glycidyl ether (PGE) from epoxy resin systems. Chem Res Toxicol.

[45] Bergstrom MA, Luthman K, Karlberg AT (2007). Metabolic epoxidation of an alpha,beta-unsaturated oxime generates sensitizers of extreme potency. Are nitroso intermediates responsible?. Chem Res Toxicol.

[46] Shimizu T (2003). Impaired contact hypersensitivity in macrophage migration inhibitory factor-deficient mice. Eur J Immunol.

[47] Pichler WJ (2005). Direct T-cell stimulations by drugs–bypassing the innate immune system. Toxicology.

[48] Martin SF (2010). T-cell recognition of chemicals, protein allergens and drugs: towards the development of *in vitro* assays. Cell Mol Life Sci.

[49] Pichler WJ (2003). Delayed drug hypersensitivity reactions. Ann Intern Med.

[50] Yun J, Cai F, Lee FJ, Pichler WJ (2016). T-cell-mediated drug hypersensitivity: immune mechanisms and their clinical relevance. Asia Pac Allergy.

[51] Sieben S, Kawakubo Y, Al Masaoudi T, Merk HF, Blomeke B (2002). Delayed-type hypersensitivity reaction to paraphenylenediamine is mediated by 2 different pathways of antigen recognition by specific alphabeta human T-cell clones. J Allergy Clin Immunol.

[52] Pichler WJ (2008). The p-i Concept: Pharmacological Interaction of Drugs With Immune Receptors. World Allergy Organ J.

[53] Diamond DJ (1991). Major histocompatibility complex independent T cell receptor-antigen interaction: functional analysis using fluorescein derivatives. J Exp Med.

[54] Pichler WJ, Daubner B, Kawabata T (2011). Drug hypersensitivity: flare-up reactions, cross-reactivity and multiple drug hypersensitivity. J Dermatol.

[55] Rajasekaran D (2016). Macrophage Migration Inhibitory Factor-CXCR4 Receptor Interactions: Evidence for Partial Allosteric Agonism in Comparison with CXCL12 Chemokine. J Biol Chem.

[56] Morand EF (2002). Macrophage migration inhibitory factor in rheumatoid arthritis: clinical correlations. Rheumatology (Oxford).

[57] de Jong YP (2001). Development of chronic colitis is dependent on the cytokine MIF. Nat Immunol.

[58] Bernhagen J (1993). MIF is a pituitary-derived cytokine that potentiates lethal endotoxaemia. Nature.

[59] Emonts M (2007). Association between high levels of blood macrophage migration inhibitory factor, inappropriate adrenal response and early death in patients with severe sepsis. Clin Infect Dis.

[60] Mitchell RA (2004). Mechanisms and effectors of MIF-dependent promotion of tumourigenesis. Cell Signal.

[61] Bucala R, Donnelly SC (2007). Macrophage migration inhibitory factor: a probable link between inflammation and cancer. Immunity.

[62] O’Reilly C, Doroudian M, Mawhinney L, Donnelly SC (2016). Targeting MIF in Cancer: Therapeutic Strategies, Current Developments and Future Opportunities. Med Res Rev.

[63] Brocks T (2017). Macrophage migration inhibitory factor protects from nonmelanoma epidermal tumors by regulating the number of antigen-presenting cells in skin. FASEB J.

[64] Heise R (2012). Expression and function of macrophage migration inhibitory factor in the pathogenesis of UV-induced cutaneous nonmelanoma skin cancer. Photochem Photobiol.

