# Supplementary material for: The Fate of a Hapten - From the Skin to Modification of Macrophage Migration Inhibitory Factor (MIF) in Lymph Nodes
Source: Sci Rep. 2018 Feb 13;8:2895. doi: 10.1038/s41598-018-21327-8 (PMC5811565; doi:10.1038/s41598-018-21327-8)
Supplement: Supplementary file 1 — Supplementary Information [file 41598_2018_21327_MOESM1_ESM.pdf]

SUPPLEMENTARY MATERIAL FOR:

The Fate of a Hapten - From the Skin to Modification of Macrophage Migration Inhibitory Factor (MIF) in Lymph Nodes

Isabella Karlsson[1,*], Kristin Samuelsson[2], Carl Simonsson[2], Anna-Lena Stenfeldt[2], Ulrika Nilsson[1], Leopold L Ilag[1], Charlotte Jonsson[2], and Ann-Therese Karlberg[2]

[1] Department of Environmental Science and Analytical Chemistry, Stockholm University, Sweden
[2] Department of Chemistry and Molecular Biology, Dermatochemistry, University of Gothenburg, Sweden

* Correspondence: Isabella Karlsson, Department of Environmental Science and Analytical Chemistry, Stockholm University, SE-106 91 Stockholm, Sweden. E-mail: isabella.karlsson@aces.su.se

TABLE OF CONTENTS

ANIMALS **3**

ASSESSMENT OF SKIN SENSITISATION POTENTIAL OF RHODAMINE B IN THE LOCAL LYMPH NODE ASSAY **3**

Table S1. LLNA responses for Rhodamine B *3*

TREATMENT OF MICE AND TISSUE COLLECTION FOR FLUOROPHORE DISTRIBUTION STUDIES **4**

LSCM OF EAR- AND LYMH NODE SECTIONS **4**

DETECTION OF FLUORESCENT DRAINING LYMH NODE CELLS USING FLOW CYTOMETRY **5**

Table S2. Lymph node cellularity[1] and proportion of fluorescent cells. *5*

WESTERN BLOT DETECTION OF THE POTENTIAL TRITC TARGET-PROTEIN MIF **6**

Figure S1. Fluorescence detection of TRITC-bound proteins and western blot with MIF antibody *6*

DETECTION OF TRITC-MODIFIED AND NON-MODIFIED MIF-PEPTIDE (PMFIVNTNVPR) IN LYMPH NODE FRACTIONS USING UPLC-qToF **7**

Figure S2. Chromatogram and spectra of the N-terminal peptide of MIF *9*

Figure S3. Standard curve for PMFIVNTNVPR *10*

REFERENCES **11**

ANIMALS

Female CBA/Ca mice (NOVA-SCB, Sollentuna, Sweden) 8-12 weeks of age were used. The mice were housed in cages with HEPA-filtered airflow under conventional conditions in light-, humidity-, and temperature-controlled rooms. The regional ethics committee, Jordbruksverket, approved all experimental protocols and the animal procedures were carried out in accordance with the approved guidelines.

ASSESSMENT OF SKIN SENSITISATION POTENTIAL OF RHODAMINE B IN THE LOCAL LYMPH NODE ASSAY

The local lymph node assay (LLNA)[1-3] was used to predict the sensitization potential of tetraethylrhodamine (Rhodamine B), a structural analogue of TRITC that lacks the reactive NCS group. Briefly, mice were divided into groups of 3, and 25 µl of the test compound was applied on the dorsum of each ear for 3 consecutive days. Rhodamine B was dissolved in dimethyl sulfoxide (DMSO) and tested at 1×10^{-3} % (w/v), 0.010%, 0.10%, 1.0%, 5.0% (21 µM, 210 µM, 2.1 mM, 21 mM). A control group received vehicle alone (i.e. DMSO). Three days after the last exposure, 20 µCi [methyl-^{3}H] thymidine was injected intravenously in the tail vein. Five hours later the mice were sacrificed and draining lymph nodes from each treatment group were excised and pooled. Single cell suspensions were prepared and cells were incubated over night at 4 °C in 5% (w/v) trichloroacetic acid (TCA). The next day, the cells were centrifuged and resuspended in TCA and scintillation cocktail (EcoLume, INC Radiochemicals). The [methyl-^{3}H] thymidine incorporation was measured by β-scintillation counting with a Beckman LS 6000TA instrument. Results are expressed as mean disintegrations per minute (DPM)/pooled lymph node and as a stimulation index (SI). The SI is the ratio of DPM/lymph node for the test group compared with the control group. A test compound is considered positive if it at one or more concentrations gives an SI greater than 3. EC3 values (the estimated concentration required to induce an SI of 3) are calculated by linear interpolation. Rhodamine B failed to give an SI > 3, which classifies Rhodamine B as a non-sensitizer in the tested concentrations.

Table S1. LLNA responses for Rhodamine B

**Concentration**		**DPM/node**	**SI**
%	mM		
Vehicle*		669	
0.0010	0.021	590	0.88
0.020	0.21	643	0.96
0.10	2.1	611	0.91
1.0	21	528	0.79
5.0	105	505	0.75
**EC3**			**not applicable**

*DMSO

TREATMENT OF MICE AND TISSUE COLLECTION FOR FLUOROPHORE DISTRIBUTION STUDIES

Groups of mice were topically exposed to 25 µl of TRITC (0.25% (w/v), 5.6 mM, 12 mice or 0.50%, 11 mM, 6 mice), Rhodamine B (0.54%, 11 mM, 6 mice), or vehicle (acetone:dibutyl phtalate, 1:1, 18 mice), on the dorsum of the ears for three consecutive days. Eighteen hours after the last exposure, the mice were sacrificed and the ears and the auricular lymph nodes were excised. For histological analyses, ears (after being wiped with PBS) and lymph nodes were embedded in TissueTek (Sakura Finetek, Zoeterwoude, Netherlands), snap frozen in 96% ethanol cooled with dry ice, and stored at -70 °C until used. For analysis of cells and proteins, lymph node single cell suspensions were immediately prepared by pooling lymph nodes from the same treatment group and mashing them through cell strainers (Falcon, BD labware, 70 µm pore size) in PBS. Further processing for flow cytometry or protein analysis is described in the respective subsections in the article or below.

LSCM OF EAR- AND LYMH NODE SECTIONS

Frozen ears and lymph nodes were sectioned into 10 µm and 14 µm thick samples, respectively, on a cryostat (Leica Microsystems, Wetzlar, Germany). Sections were placed on SuperFrost Plus Slides (Menzel-Gläser, Braunschweing, Germany) and dried over night at room temperature in the dark. Next day, slides were fixed in ice cold acetone (4 °C) for 15 min, dried for 15-30 min, washed in PBS for 3×5 min, in tap-water for 2×10 min, and in ultra-pure water (Purelab Ultra, Ninolab, Sweden) for 1×10 min. Fixed slides were mounted with Fluoromount mounting media (Sigma-Aldrich Chemie GmbH), and sealed over night with Clarion mounting media (Sigma-Aldrich Chemie GmbH) at room temperature. The slides were stored dark at 4 °C until analysis. Imaging of ear and lymph node thin sections was performed using an LSCM 510 Meta system (Zeiss, Jena, Germany), at the Centre for Cellular Imaging, University of Gothenburg, Sweden. Ear samples were imaged using an Achroplan 40x/0.8w objective (Zeiss, Jena, Germany), and lymph nodes using a Plan-Neufluar 10x/0.3 objective (Zeiss, Jena, Germany). The excitation source was an HeNe 543 nm laser and the emission was separated from excitation light using a 560 LP filter.

DETECTION OF FLUORESCENT DRAINING LYMH NODE CELLS USING FLOW CYTOMETRY

Single cell suspensions of lymph node cells were washed with PBS, and diluted to 10^6 cells ml^{-1} in PBS. The proportion of fluorescent lymph node cells from mice exposed to TRITC (11 mM or 5.6 mM), Rhodamine B (11 mM), or vehicle was measured by a FACSArray flow cytometer (BD Biosciences) equipped with a 532 nm excitation laser. The distribution of TRITC in different lymph node cell populations were investigated using typical markers for B-cells (CD19), T-cells (CD3ε), and dendritic cells (CD11c). Briefly, Fc-receptors were blocked by 1 µg mouse Fc-block (BD Biosciences) per million cells, thereafter the cells were stained with allophycocyanine (APC) labeled antibodies; rat anti-mouse CD19 (clone 6D5) (0.5 µg/million cells), armenian hamster anti-mouse CD3ε (clone 145-2C11), and CD11c (clone N418) (BioLegend) (0.25 µg and 0.5 µg per million cells, respectively). Unbound antibody was rinsed off before flow cytometry analyses. The BD FACSArray system software was used for the data analysis.

Table S2. Lymph node cellularity[1] and proportion of fluorescent cells.

Compound[2]	Average number of cells/lymph node (x 10^6)[3]	Fluorescent cells (%)
TRITC	6.2	77
Rhodamine B	1.5	1
Vehicle[4]	1.6	2

[1] Mice were exposed to TRITC, Rhodamine B or vehicle for three consecutive days on dorsum of both ears. Mice were sacrificed 18 h after the last application, and the lymph nodes were excised and pooled for each group and single cell suspensions were prepared.

[2] The concentration of TRITC and Rhodamine B was 11 mM.

[3] The cells were counted in a Bürkner chamber and the average number of cells/lymph node was calculated.

[4] Acetone:dibutyl phthalate (1:1).

WESTERN BLOT DETECTION OF THE POTENTIAL TRITC TARGET-PROTEIN MIF

The presence of MIF in gel-separated proteins derived from lymph node cells was confirmed with western blot. Lymph node cell proteins were separated with gel electrophoresis as described in the article. Separated proteins were transferred to a polyvinylidene fluoride (PVDF) membrane (Millipore) and TRITC-fluorescent proteins were detected with a fluorescence laser scanner (Typhoon Variable Mode Imager 9410) before immunostaining with MIF antibody. The PVDF membrane was blocked by blocking buffer at room temperature for 40 min and incubated for 60 min with the polyclonal rabbit anti-rat/mouse MIF antibody (ab7207, abcam). After washing, the membrane was incubated for 90 min with the secondary goat anti-rabbit IgG antibody, conjugated with DyLight 488 (35553, Thermo Scientific). Antibody marked proteins were detected with a Typhoon Variable Mode Imager 9410 using a short pass filter and excitation/emission wavelength of 488/526 nm.

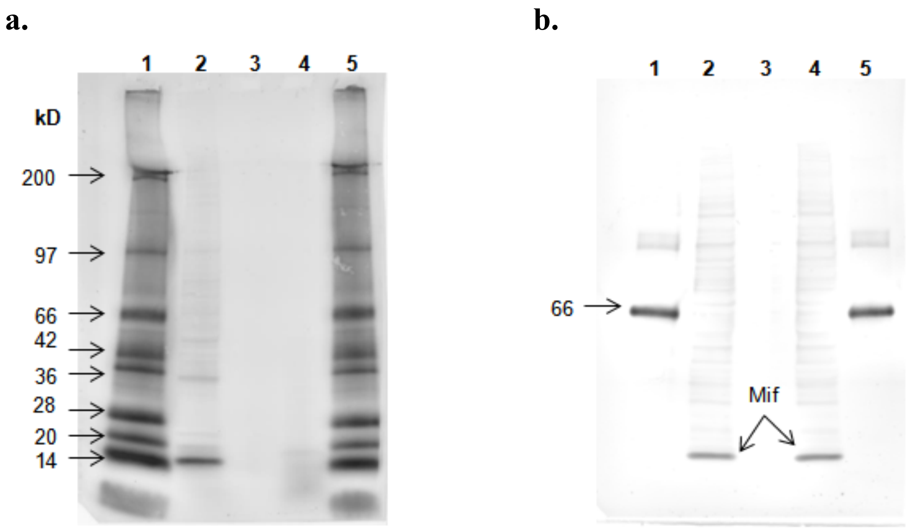

Figure S1. Fluorescence detection of TRITC-bound proteins and western blot with MIF antibody

Lymp node cell proteins were isolated and pooled from mice exposed to vehicle (acetone:dibutyl phtalate, n=10) or TRITC (5.6 mM, n=6) on the dorsum of the ears for three consecutive days. Proteins were separated by 1D SDS-PAGE and blotted on a PVDF membrane. (a) TRITC-fluorescent proteins on the membrane were visualized in a fluorescence laser scanner at excitation/emission wavelength 532/580 nm. Dylight 549/649 was used as a molecular standard (well 1 and 5). Fluorescent protein bands were clearly visualized in samples from TRITC-exposed mice (well 2). No fluorescence could be detected in samples from vehicle-exposed mice (well 4). (Well 3 was left empty to avoid carry over.) (b) Immunoblotting with a primary rabbit anti-rat/mouse MIF antibody followed by a secondary goat anti-rabbit IgG antibody conjugated with DyLight 488 and scanning at excitation/emission wavelength of 488/526 nm. An approximate co-localization of MIF (b, well 2 and 4) with the most intense TRITC-fluorescent band (a, well 2) around 14 kD was observed.

DETECTION OF TRITC-MODIFIED AND NON-MODIFIED MIF-PEPTIDE (PMFIVNTNVPR) IN LYMPH NODE FRACTIONS USING UPLC-qToF

To identify TRITC-bound MIF and non-modified MIF in the lymph node single cell suspensions from TRITC-treated mice, the 10-15 kDa section from the gel electrophoresis separation of the proteins was divided into three pieces (Figure 3): **A** – the piece just below the fluorescent band, **B** – the fluorescent band, and **C** – the piece just above the fluorescent band. In-gel digestion, including reduction with DTT and alkylation with IAA, was performed using Trypsin/Lys-C mix (Promega) according to the manufacturer's instructions. Thereafter, the extracted peptides were concentrated to 30% of the volume using a stream of N_2.

UPLC-qToF analysis was performed using an Aquity UPLC system (Waters Corporation) coupled to a SYNAPT G2 (Waters MS Technologies) orthogonal acceleration qToF mass spectrometer. The UPLC was equipped with an Aquity CSH C18 column (150 × 2.1 mm i.d., particle size 1.7 μm, Waters Corporation, Milford, MA). Mobile phase A consisted of 0.1% formic acid in water and mobile phase B of 0.1% formic acid in acetonitrile. Aliquots of 10 μl of sample and standard were injected onto the column and eluted with a flow rate of 0.1 ml/min and a column temperature of 40 °C. The gradient conditions used were: 0 min 5% B, 10 min 60% B, 11 min 95% B, 12 min 95% B. The system was equilibrated with 5% B for 3 min between each run. The qToF was operated in positive resolution mode with electrospray ionization. The nebulizer gas was set to 800 l/h at a temperature of 450 °C. The cone gas was set to 100 l/h and the source temperature to 120 °C. The capillary voltage was set to 3000 V and the sample cone voltage was set to 80 V. A ToF ms/ms method was used where the fixed mass was set to 865.9 or 644.3 m/z, the ms^2 was set to 50-1500 m/z, and the collision energy ramp was from 15 eV to 40 eV. To ensure mass accuracy, all analyses were acquired using LockSpray with Leucine-enkephaline (556.2771 m/z) as lock mass. The obtained chromatograms and MS spectra where processed with the smooth function in the MassLynx software (Waters Corporation). The window size was set to ±3 scans and the number of smooths to 5. The Savitzky Golay method was used for chromatograms and the mean method for MS spectra.

To confirm the identity of the non-modified and TRITC-modified peptides, synthetic reference compounds were used. A standard mixture was prepared by mixing 200 μl of PMFIVNTNVPR (0.5 mM in sodium phosphate buffer (100 mM) pH 7.5/methanol 1:1) with 50 μl of TRITC (3 mM in methanol) and 750 μl of sodium phosphate buffer pH 7.5, resulting in final concentrations of 0.1 mM and 0.15 mM for PMFIVNTNVPR and TRITC, respectively. The mixture was left at room temperature for 24 h followed by 10,000-fold dilution with ammonium bicarbonate (25 mM) pH 8.0.

a.

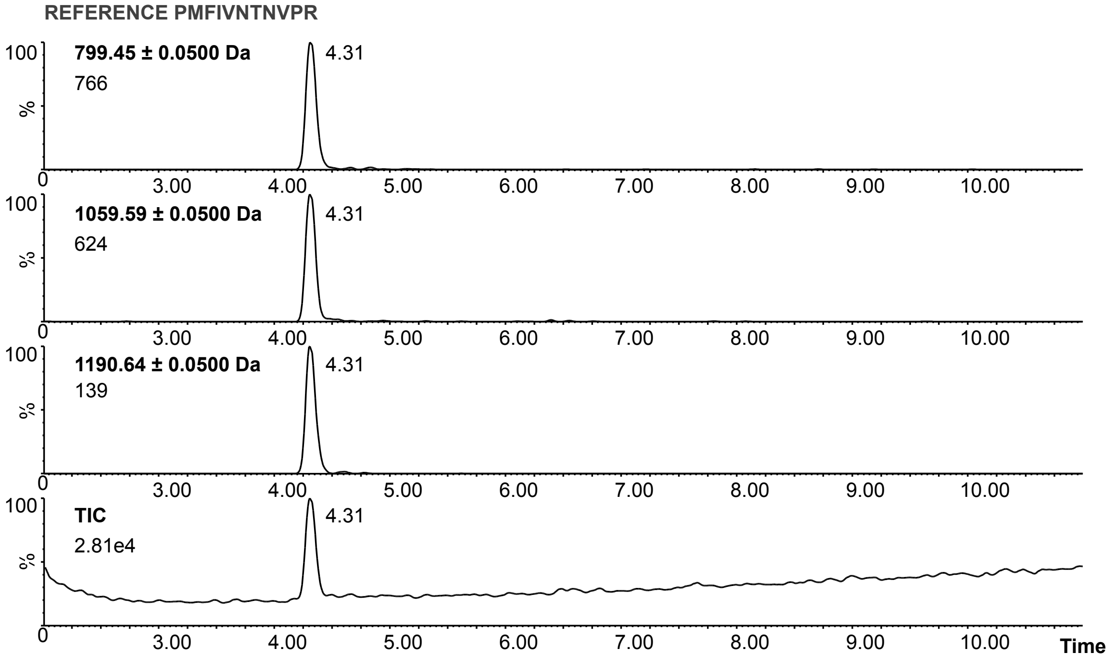
REFERENCE PMFIVNTNVPR
799.45 ± 0.0500 Da
766
1059.59 ± 0.0500 Da
624
1190.64 ± 0.0500 Da
139
TIC
2.81e4
4.31
100
%
0
3.00
4.00
5.00
6.00
7.00
8.00
9.00
10.00
Time

b.

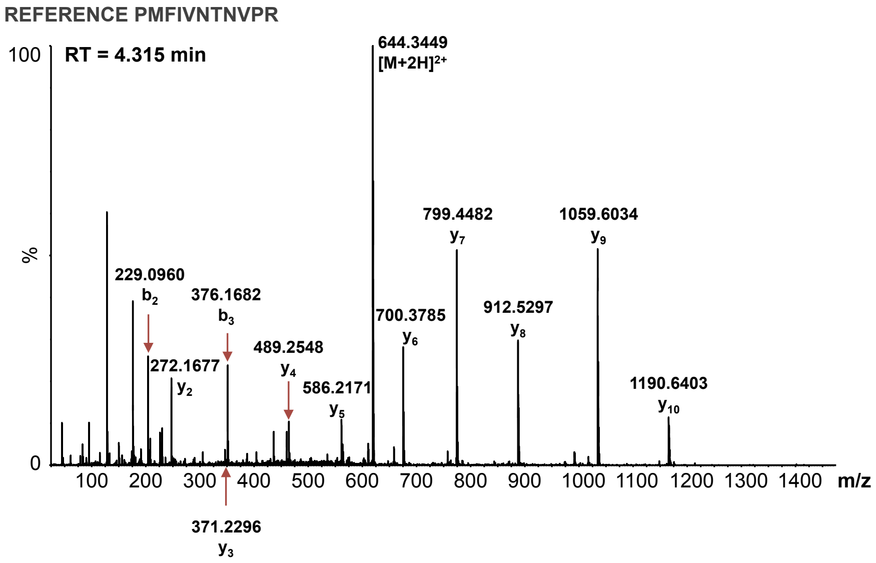
REFERENCE PMFIVNTNVPR
RT = 4.315 min
644.3449
[M+2H]2+
229.0960
b2
376.1682
b3
272.1677
y2
489.2548
y4
586.2171
y5
700.3785
y6
799.4482
y7
912.5297
y8
1059.6034
y9
1190.6403
y10
371.2296
y3
100
%
0
100 200 300 400 500 600 700 800 900 1000 1100 1200 1300 1400
m/z

c.

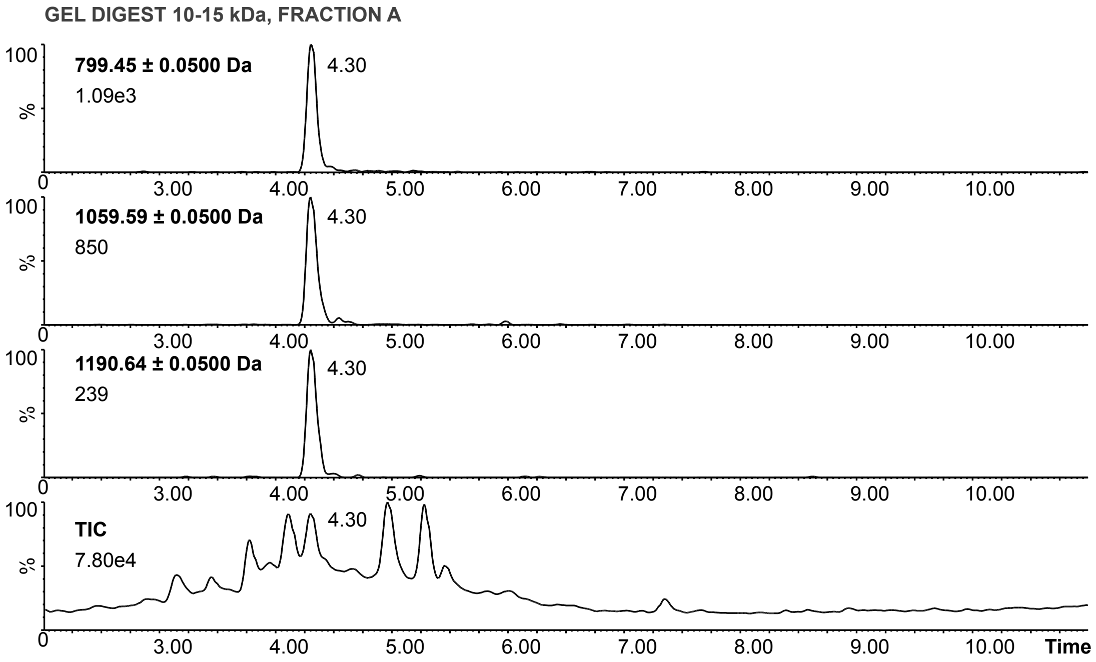

d.

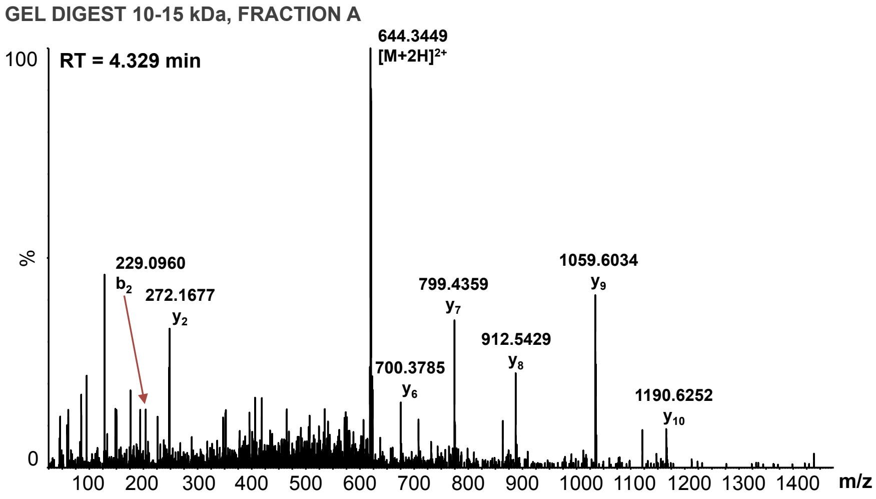

Figure S2. Chromatogram and spectra of the N-terminal peptide of MIF

Chromatograms and spectrum of the reference compound (the N-terminal MIF peptide PMFIVNTNVPR) and fraction A from lymph node cell proteins isolated from TRITC-exposed mice and separated with 1D SDS-PAGE. The selected bands (see Figure 3) were excised, digested and analyzed with UPLC-qToF. A reference compound was prepared to confirm the identity of the peptide. The qToF was operated in positive resolution mode with electrospray ionization. A qToF ms/ms method was used where the fixed mass was set to 644.3, the ms^2 was set to 50-1500 m/z, and the collision energy ramp was from 15 eV to 40 eV. (a) Chromatogram of reference compound (the extracted chromatograms correspond to fragment y_{10}, y_9, and y_7), (b) spectrum of reference compound, (c) chromatogram of fraction A (the extracted chromatograms correspond to fragment y_{10}, y_9, and y_7), and (d) spectrum of fraction A.

To obtain an approximation of the concentrations of PMFIVNTNVPR and TRITC-PMFIVNTNVPR in the reference sample and the excised gel pieces, a standard curve of PMFIVNTNVPR was created (Fig. S3). To estimate the concentration of TRITC-PMFIVNTNVPR in the reference sample it was assumed that all depletion of PMFIVNTNVPR was due to reaction with TRITC. According to the standard curve, the concentration PMFIVNTNVPR in the reference sample was around 5 nM, i.e. half of the initial amount of PMFIVNTNVPR remained. It was therefore estimated that the concentration of TRITC-modified PMFIVNTNVPR (mixture of isomers) in the reference mixture was also 5 nM.

The signal of PMFIVNTNVPR in fraction A was determined to be around 7-8 nM while the concentration of TRITC-PMFIVNTNVPR in fraction B, by comparison to the reference mixture, was estimated to be around 2-3 nM. Thus, the portion of modified MIF in the lymph nodes would be around 20-30% of the combined amount of MIF, i.e non-modified MIF (7-8 nM) plus TRITC modified-MIF (2-3 nM).

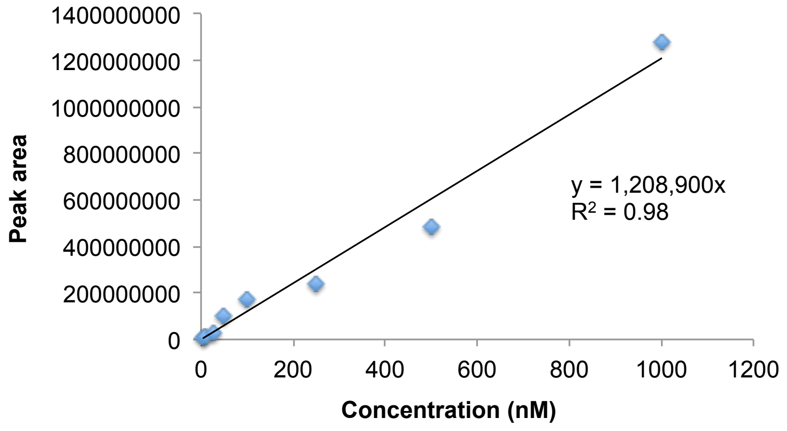

Figure S3. Standard curve for PMFIVNTNVPR

Peak areas of the peptide PMFIVNTNVPR were plotted against concentrations ranging from 5 nM to 1000 nM. By setting the intercept to zero, the obtained k value was 1,208,900 and the R^2 value was 0.98.

REFERENCES

1 Gerberick, G. F., Ryan, C. A., Dearman, R. J. & Kimber, I. Local lymph node assay (LLNA) for detection of sensitization capacity of chemicals. *Methods* **41**, 54-60, doi:10.1016/j.ymeth.2006.07.006 (2007).

2 Basketter, D. A. *et al.* Local lymph node assay - validation, conduct and use in practice. *Food Chem Toxicol* **40**, 593-598 (2002).

3 Kimber, I. & Weisenberger, C. A murine local lymph node assay for the identification of contact allergens. Assay development and results of an initial validation study. *Arch Toxicol* **63**, 274-282 (1989).
